# Case Report: A novel strategy of endoscopic full-thickness resection: single traction combined with pre-clamping

**DOI:** 10.3389/fmed.2025.1581544

**Published:** 2025-07-02

**Authors:** Jinguo Liu, Jing Xu, Li Wang, Xiaojiang Gu, Xuesong Zhang, Zhou Zhang, Chi Zhang, Liangliang Yu

**Affiliations:** ^1^Department of Endoscopy Center, Sir Run Run Shaw Hospital, Zhejiang University, Hangzhou, China; ^2^Department of Pathology, Sir Run Run Shaw Hospital, Zhejiang University, Hangzhou, China; ^3^Department of Surgery, Huangshi Traditional Chinese Medicine Hospital, Hubei Chinese Medical University, Huangshi, China

**Keywords:** endoscopic full-thickness resection, submucosal tumor, single traction, pre-clamping, en bloc resection

## Abstract

Endoscopic full-thickness resection (EFTR) is an effective, economical, and minimally invasive technique for submucosal tumors (SMTs). However, technical complexity and prolonged operative time remain significant limitations. This study introduces a refined EFTR strategy termed “single traction combined with pre-clamping” in a cohort of 10 patients. The proposed technique demonstrates reduced procedural duration, technical difficulty, and intraoperative risks compared to the conventional approach.

## Introduction

Endoscopic full-thickness resection (EFTR) has emerged as a therapeutic cornerstone for SMTs originating from the muscularis propria layer (1, 2). Despite its efficacy, post-resection wound closure remains technically demanding (3, 4), with serosal surface bleeding and inadvertent tumor displacement into the abdominal cavity posing critical challenges. To address these limitations, we present an innovative EFTR protocol integrating single traction with pre-clamping.

## Case description

**FIGURE 1:**
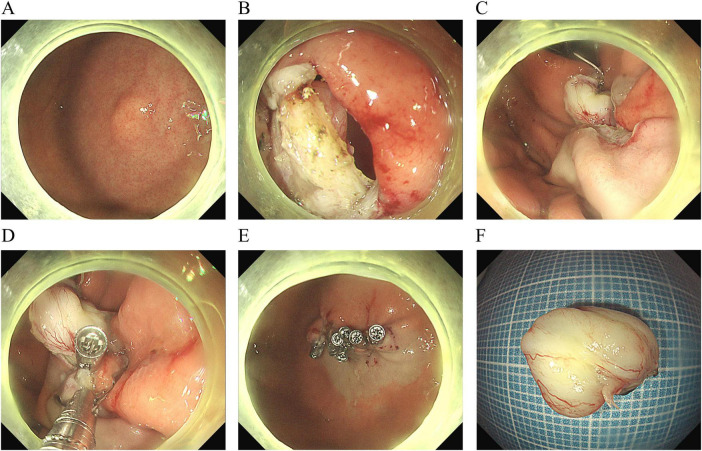
The procedural steps of endoscopic full-thickness resection with “single traction combined with pre-clamping.” **(A)** A submucosal tumor in the gastric fundus. **(B)** The exposed full-thickness incision. **(C)** Single traction using the dental floss. **(D)** Pre-clamping with three titanium clips. **(E)** Closure of the wound. **(F)** Macroscopic appearance of the resected tumor (measuring 15.0 × 11.0 mm).

Ten patients (3 males, 7 females; mean age 61.7 years) with SMTs (gastric fundus: 8, gastric body: 1, duodenal bulb: (1) underwent EFTR using the novel technique (Table 1). Illustrative Case: A 57-year-old female with a gastric fundus SMT (Figure 1A) underwent EFTR after obtaining informed consent. Key procedural steps included: (1) Full-thickness incision to expose the tumor (Figure 1B). (2) Traction using the dental floss anchored with a titanium clip (Figure 1C). (3) Pre-clamping with three titanium clips (Figure 1D, Supplementary Video 1). (4) En bloc tumor resection with complete wound closure (Figure 1E). The operative time from full-thickness incision to wound closure took about

**TABLE 1:** Characteristics of ten patients undergoing EFTR with the “single traction combined with pre-clamping” technique.

Number	Sex	Age	Location	Size, cm	Procedure time, minutes	Follow up, months	Traction combined with pre-clamping
1	F	57	Gastric fundus	1.5 × 1.1	48	6	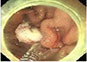
2	F	68	Duodenal bulb	2.0 × 1.5	83	6	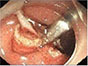
3	F	72	Gastric fundus	1.5 × 1.0	25	5	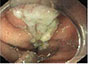
4	M	52	Gastric fundus	1.2 × 0.9	56	5	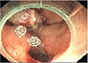
5	F	50	Gastric fundus	0.9 × 0.6	36	5	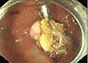
6	F	72	Gastric fundus	1.1 × 1.0	60	5	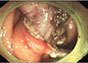
7	M	68	Gastric fundus	1.5 × 1.0	123	6	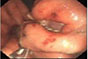
8	M	56	Gastric body	1.6 × 1.0	46	5	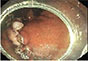
9	F	62	Gastric fundus	0.7 × 0.6	54	3	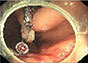
10	F	60	Gastric fundus	2.0 × 1.8	55	3	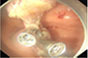

only 12 min, and the tumor was pulled out (Figure 1F). The patient was discharged on the third day without any complications. Postoperative pathology showed a low-grade stromal tumor (Figures 2A–D).

**FIGURE 2:**
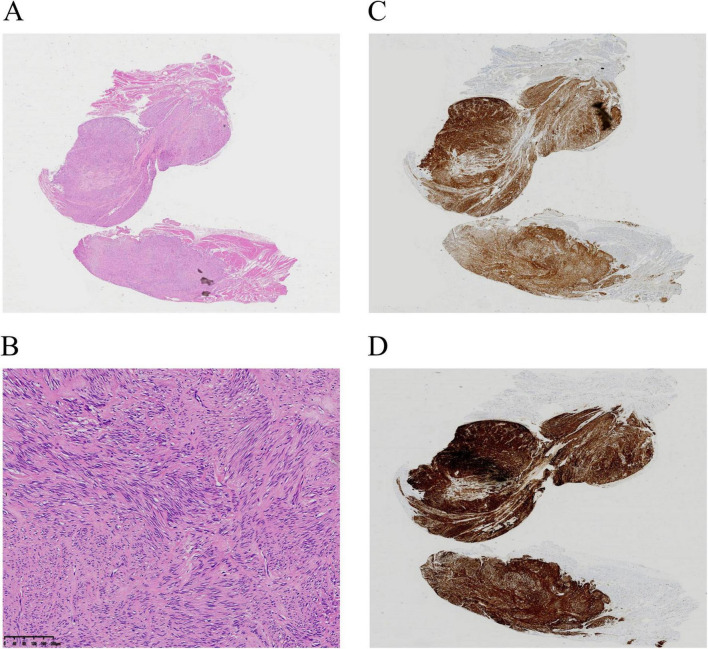
Pathologic examination. **(A,B)** Hematoxylin–eosin. Magnification: ×20 **(A)**, ×100 **(B)**. **(C,D)** Immunohistochemistry showing positive areas for CD117 **(C)** and DOG-1 **(D)**.

The size of the tumors ranged from 0.7 × 0.6 cm to 2.0 × 1.8 cm, with procedural durations spanning 25–123 min (Table 1). None of the patients experienced postoperative bleeding or infection. Pathology in all patients showed a low-grade stromal tumor. No recurrence occurred during the follow-up period (3–6 months).

## Discussion

While most SMTs exhibit benign behavior, malignant transformation potential persists in up to 13% of muscularis propria-originating lesions (5). EFTR is an effective, economical, and minimally invasive technique for SMTs originating from deep muscularis propria layer (4, 6). The traction method facilitated wound edge inversion, enabling effective pre-clamping and mitigating serosal bleeding risks. Proactively identifying and managing exposed serosal hemorrhagic vessels might effectively minimize the risk of postoperative bleeding. In addition, controlled traction maneuvers facilitated optimal tumor retraction into the gastric lumen, enabling direct visualization and en bloc resection under endoscopic surveillance.

Traction assistance is a practical technique for EFTR without severe perioperative adverse events. Double traction assistance was used in EFTR for the resection of a gastric submucosal tumor (4). Prior studies report operative times of 28–89 min using clip-assisted traction (7) and 25–130 min with snare-assisted methods (8), both without severe adverse events. Notably, snare traction demonstrated significantly shorter operative durations and a lower incidence of intraoperative bleeding versus conventional EFTR (53.6 ± 16.6 min vs 67.7 ± 33.4 min, *P* < 0.001) (9). In addition, traction assistance was used to remove an embedded gastric fishbone (10).

Our technique achieved comparable efficiency (25–123 min), with variability attributable to the size and location of the tumor, as well as the proficiency level of the endoscopist. The EFTR case with 123 min was performed by a novice operator, whereas experienced endoscopists completed the procedure in 25–83 min. Notably, when excluding the case involving the duodenal bulb, an anatomically challenging location, procedural durations were further streamlined to 25–60 min.

Clip-based traction modalities (e.g., clip-line, clip-snare, clip-rubber band) and non-clip alternatives (external forceps, gravity-based traction) have been extensively utilized (11–15). Our protocol uniquely integrates single traction with pre-clamping, offering dual advantages of time reduction and risk mitigation.

## Data Availability

The original contributions presented in this study are included in this article/Supplementary material, further inquiries can be directed to the corresponding author.

## Supplementary material

Supplementary Video 1 Single traction with the dental floss and pre-clamping with three titanium clips.
